# Key Phytochemicals and Biological Functions of *Chuanxiong Rhizoma* Against Ischemic Stroke: A Network Pharmacology and Experimental Assessment

**DOI:** 10.3389/fphar.2021.758049

**Published:** 2021-12-21

**Authors:** Peng Zeng, Yao Yi, Hong-Fei Su, Chao-Yuan Ye, Yi-Wen Sun, Xin-Wen Zhou, Youming Lu, Anbing Shi, Qing Tian

**Affiliations:** ^1^ Key Laboratory of Neurological Disease of National Education Ministry, Department of Pathology and Pathophysiology, School of Basic Medicine, Tongji Medical College, Huazhong University of Science and Technology, Wuhan, China; ^2^ Department of Biochemistry and Molecular Biology, School of Basic Medicine, Tongji Medical College, Cell Architecture Research Institute, Huazhong University of Science and Technology, Wuhan, China

**Keywords:** *Chuanxiong Rhizoma*, ischemic stroke, network pharmacology, key phytochemicals, pathophysiology

## Abstract

Presently, the treatment options for ischemic stroke (IS) are limited due to the complicated pathological process of the disease. *Chuanxiong Rhizome* (CR), also known as *Conioselinum anthriscoides* “Chuanxiong” (rhizome), is the most widely used traditional Chinese medicine for treating stroke. This study aimed to uncover the key phytochemicals and biological functions of CR against IS through a network pharmacology approach combining with IS pathophysiology analysis. We employed permanent unilateral common carotid artery ligation to construct a mouse model of global cerebral ischemia and found that cerebral ischemia injuries were improved after 7 days of gavage treatment of CR (1,300 mg/kg/day). CR exerts protective effects on neurons mainly by acting on targets related to synaptic structure, synaptic function, neuronal survival and neuronal growth. A total of 18 phytochemicals from CR based on UHPLC-MS/MS that corresponded to 85 anti-IS targets. Coniferyl ferulate, neocnidilide and ferulic acid were identified as the key phytochemicals of CR against IS. Its brain protective effects involve anti-inflammatory, anti-oxidative stress, and anti-cell death activities and improves blood circulation. Additionally, the two most important synergistic effects of CR phytochemicals in treating IS are prevention of infection and regulation of blood pressure. In brain samples of Sham mice, L-tryptophan and vanillin were detected, while L-tryptophan, gallic acid, vanillin and cryptochlorogenic acid were detected in IS mice by UHPLC–MS/MS. Our findings provide a pathophysiology relevant pharmacological basis for further researches on IS therapeutic drugs.

## Introduction

Ischemic stroke (IS), which accounts for 87% of all cerebral strokes, is a leading cause of neurological morbidity and mortality worldwide (Benjamin et al., 2018). It can be induced by a variety of events, such as cardiac ischemia, occlusion of cerebral small blood vessels and carotid atherosclerosis. With the accelerated growth of the aging population (65 years and older), the incidence of IS is increasing yearly, and the overall burden is shifting to a younger population, especially in third world countries (Wang W. et al., 2017). The course of IS is broadly divided into the acute phase (from minutes to 6–12 h), sub-acute phase (from 6–12 h to days) and recovery phase (after more than 3  weeks). A variety of complex molecular and cellular interactions drive the pathophysiological effects of IS. Reduced blood flow to the brain causes stress, cell death (necrosis and apoptosis) and loss of neuronal function. Inflammation, oxidative stress, acidosis, overload of intracellular calcium, excitotoxicity, free radical injury, cytokine injury, complement activation, blood-brain barrier (BBB) impairment and activation of astrocytes and microglia are also key events contributing to IS pathology.

Treatment options for IS are currently limited. The recanalization of blood flow in acute IS relies on the administration of tissue plasminogen activator (t-PA) and mechanical thrombolytic technology in time, which have shown some benefits in helping patients recover from IS. However, the shortcoming of these treatments is that reperfusion can lead to the production of highly harmful reactive oxygen species (ROS) and oxidative stress. This is the major cause of reperfusion damage after ischemic injury. Oxidative stress causes apoptosis, autophagy, and necrosis in the brain. The ischemia time is a good biomarker for brain tissue viability. Clinically, the therapeutic window for tPA treatment is very limited, as tPA must be administered within 6 h of the onset of symptoms (van der Worp and van Gijn, 2007). Additionally, tPA may promote cerebral hemorrhage and anaphylaxis (Donnan et al., 2008). Therefore, there is still a great need to find therapeutic agents for IS during ischemia and recanalization, extend the therapeutic window and further improve clinical outcomes.

In clinical practice, Chinese herbal medicine has been shown to be effective in treating IS (Yuan et al., 2008). From nearly 1,000 anti-stroke Chinese medicine prescriptions, 192 anti-stroke herbs were identified (Liu et al., 2017). *Chuanxiong Rhizome* (CR), also known as *Conioselinum anthriscoides* “Chuanxiong” (rhizome), an herb first recorded in the *Shennong’s Classic of Materia* (simplified Chinese: 神农本草经), is the most frequently used herb for stroke (509 times) (Liu et al., 2017). CR is also used in treating cardiovascular diseases, respiratory diseases, pain, and trauma (Chen et al., 2018). Tetramethylpyrazine, a natural alkaloid extracted from CR, has been used extensively for the treatment of IS (Lin et al., 2021; Zhu et al., 2021). Z-ligustilide, a major component of CR, can significantly decrease the infarct volume, and mitigate neurological dysfunction in rats with middle cerebral artery occlusion (MCAO) (Li et al., 2017). CR contains numerous phytochemicals, such as a variety of aromatic acids, phthalides and alkaloids (Zhang et al., 2019; Wang et al., 2020). Analyzing and summarizing the effective phytochemicals of CR and their mechanism of action against stroke is of great significance for understanding the key intervention links in the treatment of stroke and the development of related drugs.

As a systematic research method (Hopkins, 2008; Zeng et al., 2021a; 2021b), network pharmacology can be used to study the effects of drugs in a systematic and comprehensive way. The aim of this research was to investigate the anti-IS effects of key active phytochemicals of CR and determine the anti-IS mechanism of CR based on network pharmacology research and pathophysiological changes in IS. To improve the quality of collected data, the bioactive phytochemicals of CR were obtained from a recent study based on ultra-HPLC with triple quadrupole MS (UHPLC-MS/MS) (Wang et al., 2020). Based on the pathophysiology of IS and 18 bioactive phytochemicals of CR corresponding to 85 anti-IS targets, CR was found exert neuroprotective, anti-inflammatory, anti-oxidative stress, anti-cell death and improved blood circulation effects to protect the brain. Coniferyl ferulate, neocnidilide and ferulic acid were suggested as the key phytochemicals of CR against IS. Moreover, CR phytochemicals synergistically prevent infection and regulate blood pressure in treating IS. In brain of CR treated IS mice, four components of CR, e.g., L-tryptophan, gallic acid (GA), vanillin and cryptochlorogenic acid (CA), were detected. The mechanisms by which these phytochemicals antagonize IS induced brain damage through intracerebral and extracerebral pathways require more in-depth and systematic studies.

## Materials and Methods

### Determination of the Main Phytochemicals of CR and ADME Evaluation

A total of 20 bioactive phytochemicals of CR were obtained from a recent study based on UHPLC-MS/MS (Wang et al., 2020). In addition, tetramethylpyrazine, a main bioactive phytochemical of CR, was also included (Lin et al., 2021; Zhu et al., 2021). Canonical SMILES of the 21 bioactive phytochemicals in CR were extracted from PubChem database (https://pubchem.ncbi.nlm.nih.gov/) (Kim et al., 2016). The 2-dimensional chemical structures were generated by ChemDraw Ultra 8.0 software. Lipinski’s rule of five (RO5), i.e., molecule weight (MW) < 500, number of hydrogen bond donors (Hdon) ≤ 5, number of hydrogen bond acceptors (Hacc) ≤ 10, lipid-water partition coefficient (LogP) ≤ 5 and number of rotatable bonds (Rbon) ≤ 10, has been extensively used to evaluate bioavailability based on the structures of compounds (Lipinski et al., 2001). Here, we employed the SwissADME web tool (www.swissadme.ch) (Daina et al., 2017; Zeng et al., 2021c) to evaluate the compounds according to RO5. The main phytochemicals of CR complied with RO5 were filtered out for follow-up studies. The contents of phytochemicals (mg/g) of CR were obtained from 36 CR dried rhizome samples from six different production origins. GraphPad Prism software (version 8.0, San Diego, CA, United States) was used for graphical visualization.

### Target Fishing

#### Collection of CR-Related Targets

The targets of the bioactive phytochemicals of CR were obtained using SwissTargetPrediction (http://www.swisstargetprediction.ch/) (Daina et al., 2019) based on their structures. Specifically, canonical SMILES were input into the SwissTargetPrediction, and the target species was set as *Homo sapiens*. Subsequently, target information was collected and organized using Microsoft Excel software (version 2019, Redmond, WA, United States). All software used in this study was performed on Windows (version 10).

#### Screening of Targets of CR Against IS

The disease associated with targets were retrieved from the Pathway Assembly from Literature Mining-an Information Search Tool (PALM-IST) database (http://www.hpppi.iicb.res.in/ctm/) (Mandloi and Chakrabarti, 2015) and GeneCards database (https://www.genecards.org/) (Stelzer et al., 2016) using “ischemic stroke” as a key phrase. Specifically, top 200 relevance score target genes were selected for further study in the GeneCards database. After removing duplicate targets, Venn diagrams of overlapping CR targets and disease-related targets were generated using Venny 2.1 (https://bioinfogp.cnb.csic.es/tools/venny/index.html). The common targets represented the targets of CR against IS. We further investigated the functional classification of the CR targets associated with IS using the Panther classification system (http://pantherdb.org/) (Mi et al., 2007). Sankey diagrams were plotted using OriginPro 2021 software (OriginLab Corporation, Northampton, MA, United States).

### Gene Ontology (GO) and the KEGG Pathway Enrichment Analysis

KEGG pathway database is a collection of manually drawn pathway maps of molecular interactions. GO and KEGG pathway enrichment analyses were performed using the ClusterProfiler R package (version 3.12.0 for Windows) (Yu et al., 2012). GO terms were divided into three categories: biological process (BP), cellular components (CC), and molecular function (MF). The *p* values were adjusted using a BH approach. Statistical significance was denoted if adjust *p* value <0.05. The top 10 GO terms and top 20 KEGG pathways sorted by the *p* value were visualized using an online tool (http://www.bioinformatics.com.cn/).

### Protein-Protein Interaction (PPI) Network Construction and Molecular Complex Detection (MCODE) Clustering Analysis

The PPI network of the targets was constructed using the latest version of STRING database (version 11.5, https://string-db.org/) (Szklarczyk et al., 2019) and visualized using Cytoscape software (version 3.7.1) (Shannon et al., 2003). By default, only PPIs with an interaction score exceeding the threshold of 0.4 were included. In the PPI network, degree refers to the number of other nodes directly connected to a node. The higher the degree is, the more important the node is in the PPI network. The degree values were calculated using Network Analysis (a Cytoscape plugin), and the top 10 targets ranked by degree were selected and identified as core targets.

The Cytoscape plugin MCODE (http://apps.cytoscape.org/apps/mcode) (Bader and Hogue, 2003) was employed to identify highly interconnected clusters in the PPI network. The following MCODE criteria were used for selection: degree cutoff = 2, node score cutoff = 0.2, k-core = 2, and max depth = 100. The node with the highest weighted vertex was defined as seed node (key target in the cluster) by MCODE. Moreover, a CR main component target-IS target network was constructed using Cytoscape software (version 3.7.1).

### Molecular Docking Simulations

The 3D molecular structure of CR key phytochemicals was retrieved from the PubChem database and the structure files of target proteins were acquired from the RCSB Protein Data Bank (PDB database, http://www.rcsb.org/) (Westbrook et al., 2002). Crystal structures of PTGS2 (PDB ID: 5F19), IL1B (PDB ID: 5R85), CXCL8 (PDB ID: 1ILQ) and MMP9 (PDB ID: 2OW0) were employed for molecular docking using the SwissDock web service (http://www.swissdock.ch/docking) (Grosdidier et al., 2011).

### Animals and Treatments

Male C57BL/6J mice (3-month old, *n* = 20) were from Hua Fukang Experimental Animal Center (Certificate number: SCXK (Jing) 2019-0022, Beijing, China). Ethics approval was received from the Animal Care and Use Committee of Huazhong University of Science and Technology. The animal house was kept at a constant relative humidity (55 ± 15%) and room temperature (22 ± 2°C). Mice were maintained in 12-hour-light/-dark cycle. Blood supply of brain comes from four arteries, namely two internal carotid arteries (ICAs) and two thinner vertebral arteries. Two ICAs bring about two-thirds of the blood supply into the brain. We ligated the right common carotid artery of mouse by the 5/0 surgical wire to establish a cerebral ischemia model (IS mice). Briefly, the animal was intraperitoneally anesthetized by a mixture of ketamine hydrochloride and xylazine (150 mg/ kg ketamine and 25 mg/ kg xylazine body weight). Carefully expose the right common carotid artery and permanently ligate with surgical wire. Avoid damaging the vagus nerve during the operation. Sham mouse received the same surgical exposure of right common carotid artery without ligating.

CR formula granules (ZGB 2021-111, 2 g/package) from Jiangyin Tian Jiang Pharmaceutical Co., Ltd (Jiangyin City, China) were dissolved in boiling water at a concentration of 65 mg/ ml. To evaluate the phytochemicals of CR in brain, IS mice and Sham mice were given 7-days’ CR (1,300 mg/ kg/ day) orally after surgery. The dose of CR in mice is calculated based on the clinical dose (Zhang et al., 2017). Brain samples were collected 1 h after the last gavage and stored at −80°C.

### Rotarod Test and Statistical Analysis

The rotarod test was conducted to evaluate rodent motor coordination. The mice were placed on the rotating rod for adaptive training before surgery, and the training lasted for 3 days, four rounds per day, with the rotating rod speed of 5, 10, 20, and 30 revolutions per minute (rpm) for each round of training, and the mice were trained for 180 s at each speed. The mice were tested at 10, 20, and 30 rpm in the rotating rod experiment 3 days after surgery, and the time of the mice staying on the rotating rod was recorded. In addition, the changes in body weight were recorded. Statistical analysis was performed using SPSS 12.0 (SPSS Inc., Chicago, IL). The 1-way ANOVA procedure followed by Tukey’s multiple comparisons test was used to determine the differences between groups. The level of significance was defined as *p* < 0.05.

### Triphenyltetrazolium Chloride Staining and HE Staining

TTC was obtained from Solarbio (T8170, Solarbio, Beijing, China). The mice were anesthetized and the integral brains were quickly obtained and cut into 2 mm tissue slices, stained with 2% TTC at 37°C for 20 min and soaked in 4% formaldehyde for fixation 6 h. The brain slices were arranged in order and photographed. HE staining was conducted according to routine protocols (Zeng et al., 2019). HE Staining Kit was obtained from Solarbio (G1120, Solarbio, Beijing, China). Images were acquired under a microscope (Olympus SV120, Tokyo, Japan). Cell nuclei were stained blue, while the cytoplasm and extracellular matrix were stained red in HE staining images.

### Quantitative Analysis of Key Phytochemicals of CR in Brain

The contents of 21 main bioactive phytochemicals of CR in the brain samples was analyzed by Beijing Qingxi Technology Research Institute (Beijing, China). Place 100 mg brain sample in a 15 ml centrifuge tube containing 2 ml of acetonitrile, and take 1 ml of supernatant after ultrasonication for 5 min to centrifuge at 14,000 rpm for 5 min. After the materials in the supernatant were blown dry with nitrogen, they were dissolved in 500 μL of 50% methanol aqueous solution and filtered with a 0.22 μm microporous membrane. Separation was performed on the ACQUITY UPLC HSS T3 column (2.1 × 100 mm, 1.8 µm). The flow rate was 0.3 ml/ min, the injection volume was 10 μL, and the column temperature was 35°C. Deionized water with 0.1% formic acid (phase A) and acetonitrile with 0.1% formic acid (phase B) were used as mobile phases. Data were acquired in full MS-ddMS mode using a Q Exactive Orbitrap mass spectrometer (Thermo Fisher Scientific, Bremen, Germany). The full scan (MS1) range was 100–1,200 m/ z with a resolution of 70,000, and MS2 has a resolution of 17,500. The ion source electrospray voltage was 3.2 kV, the capillary temperature was 320°C, and aux gas heater was 350°C. The sheath gas flow rate was 40 L/ min, and auxiliary gas flow rate was 15 L/ min. AGC target was set at 1e6, TopN was set at 5, and stepped normalized collision energy (NCE) of 30, 40, and 50 was used for fragmentation. By using Compound discover 3.2 software, the characteristic peaks of Raw mass spectra were extracted, and the mass deviations of the characteristic peaks for element matching, molecular formula prediction and isotope distribution matching were all set within 5 ppm. The mzcloud online database (https://www.mzcloud.org/) and the local mzVault natural products database were used to identify the characteristic peaks. The screening criteria of the positive results were mass deviation <5 ppm, coincidence isotope distribution and mzVault best match score >70.

## Results

### Main Phytochemicals of CR and Their Potential Biological Functions

**FIGURE 1:**
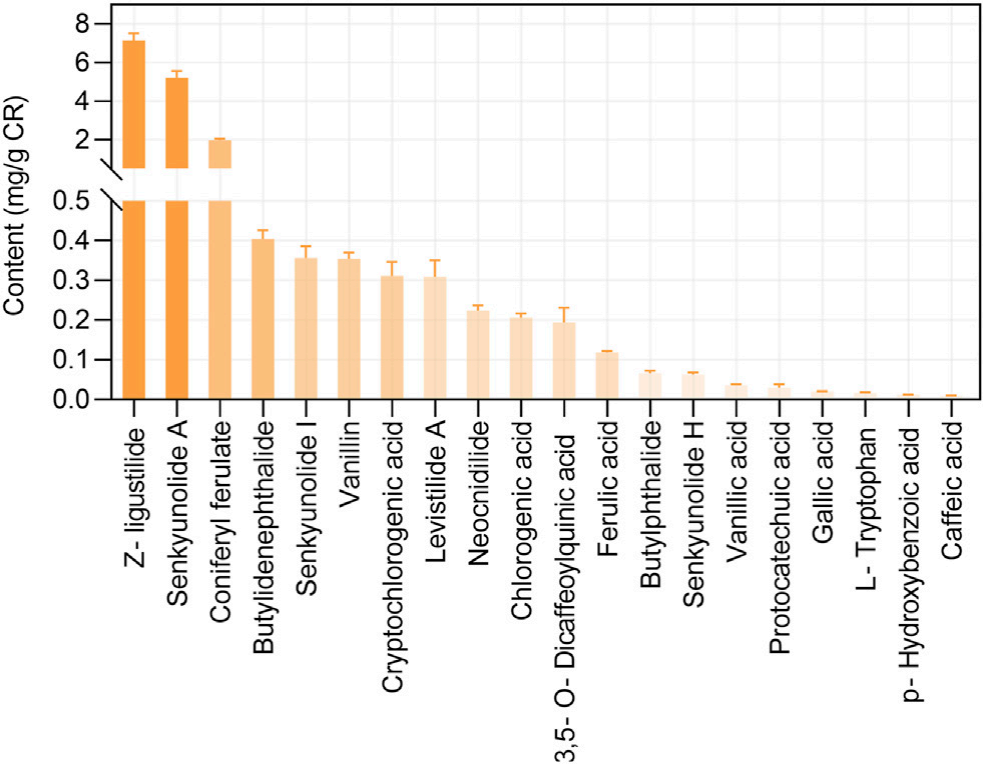
The contents of bioactive phytochemicals (mg/g) of *Chuanxiong Rhizoma* (CR). The data were retrieved from the reference (Wang et al., 2020), which used 36 CR dried rhizome samples from six different production origins in Sichuan province. The data are presented as means ± S.E.M.

This study included 21 main bioactive phytochemicals of CR, namely 12 aromatic acids, eight phthalides, and tetramethylpyrazine (Wang et al., 2020; Yin et al., 2019). According to the UHPLC-MS/MS data (Wang et al., 2020), the component with the highest content is z-ligustilide (7.16 ± 0.36 mg/g CR), followed by senkyunolide A (5.2 ± 0.36 mg/g CR) and coniferyl ferulate (1.98 ± 0.08 mg/g CR) (Figure 1). The content of tetramethylpyrazine in CR is very low (0.60 to 11.75 μg/ g CR) (Yin et al., 2018). The chemical structures of these 21 phytochemicals of CR were downloaded from the PubChem database (Kim et al., 2016). The SwissADME database (Daina et al., 2017; Zeng et al., 2021c) was employed to evaluate the phytochemicals according to RO5, a classical set of criteria used to evaluate the drug-likeness of a compound (Lipinski, 2004). Since three out of 21 phytochemicals, namely chlorogenic acid, CA and 3,5-O-Dicaffeoylquinic acid, violated RO5 (Table 1), we focused on the remaining 18 CR phytochemicals, which were consistent with RO5, in the follow-up studies.

**TABLE 1:** Pharmacological and molecular properties of the main phytochemicals in CR.

Compound	Formula	MW (g/mol)	Hdon	Hacc	Rbon	LogP
Protocatechuic acid	C_7_H_6_O_4_	154.12	3	4	1	0.65
p-Hydroxybenzoic acid	C_7_H_6_O_3_	138.12	2	3	1	1.05
L-Tryptophan	C_11_H_12_N_2_O_2_	204.23	3	3	3	0.17
Vanillic acid	C_8_H_8_O_4_	168.15	2	4	3	1.08
Gallic acid	C_7_H_6_O_5_	170.12	4	5	1	0.21
Chlorogenic acid	C_16_H_18_O_9_	354.31	6	9	5	−0.38
Caffeic acid	C_9_H_8_O_4_	180.16	3	4	2	0.93
Vanillin	C_8_H_8_O_3_	152.15	1	3	2	1.2
Ferulic acid	C_10_H_10_O_4_	194.18	2	4	3	1.36
Cryptochlorogenic acid	C_16_H_18_O_9_	354.31	6	9	5	−0.32
3,5-O-Dicaffeoylquinic acid	C_25_H_24_O_12_	516.45	7	12	9	0.76
Senkyunolide I	C_12_H_16_O_4_	224.25	2	4	2	1.17
Senkyunolide H	C_12_H_16_O_4_	224.25	2	4	2	1.18
Coniferyl ferulate	C_20_H_20_O_6_	356.37	2	6	8	3.25
Senkyunolide A	C_12_H_16_O_2_	192.25	0	2	3	2.71
Butylphthalide	C_12_H_14_O_2_	190.24	0	2	3	2.81
Z-ligustilide	C_12_H_14_O_2_	190.24	0	2	2	2.75
Butylidenephthalide	C_12_H_12_O_2_	188.22	0	2	2	2.94
Neocnidilide	C_12_H_18_O_2_	194.27	0	2	3	2.87
Levistilide A	C_24_H_28_O_4_	380.48	0	4	4	4.73
Tetramethylpyrazine	C_8_H_12_N_2_	136.19	0	2	0	0.03

MW, molecule weight; Hdon, number of hydrogen bond donors; Hacc, number of hydrogen bond acceptors; Rbon, number of rotatable bonds; LogP: lipid-water partition coefficient.

**FIGURE 2:**
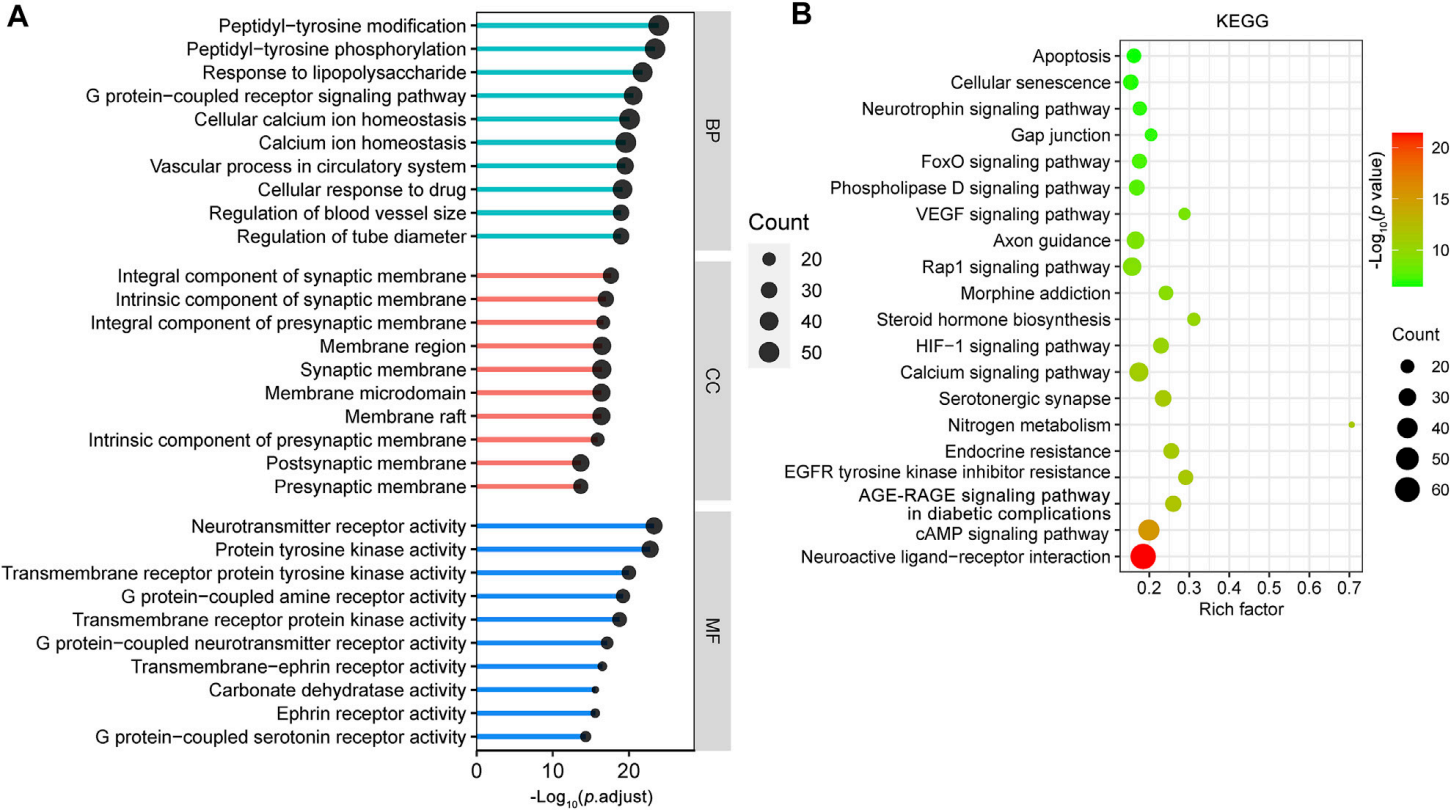
Enrichment analysis of the CR potential targets. GO functional **(A)** and KEGG pathway enrichment analysis **(B)** of 376 targets of CR main phytochemicals were identified using the ClusterProfiler package, in which the significance threshold is set as *p* value < 0.05. For the bubble plot: X-axis, rich factor (the ratio of genes in the background pathways); bubble size, the number of genes enriched; bubble color, p value. BP: biological process; CC: cellular component; MF: molecular function.

The SwissTargetPrediction database (Daina et al., 2019) was used to study the potential targets of the 18 CR phytochemicals based on their structures, and a total of 376 potential targets were obtained. To provide insight into the biological functions of these 376 targets, we performed GO and KEGG pathway enrichment analyses using the ClusterProfiler R package with the adjusted value of *p* < 0.05 (Yu et al., 2012). The GO terms included BP, CC and MF. Analysis of BP terms showed that most of the targets were related to peptidyl-tyrosine modification (GO:0018212), peptidyl-tyrosine phosphorylation (GO:0018108), response to lipopolysaccharide (LPS) (GO:0032496), G protein-coupled receptor signaling pathway (GO:0007187), cellular calcium ion homeostasis (GO:0006874) and so on. It is noteworthy that most of the CR targets were mainly localized in synaptic membranes, such as integral component of synaptic membrane (GO:0099699), intrinsic component of synaptic membrane (GO:0099240), integral component of presynaptic membrane (GO:0099056) and membrane region (GO:0098589). The top 10 enriched cell components were associated with 70 CR targets, which formed a PPI network with 69 nodes and 397 edges (Supplementary Figure S1A). Among these targets, amyloid precursor protein (APP), GRM5, SRC, GABBR1, GRM1, CNR1, HTR2A, CTNNB1, epidermal growth factor receptor (EGFR), and FYN have important roles in the PPI network (Supplementary Figure S1B). KEGG pathway analysis revealed that these targets were enriched in multiple synapse related KEGG pathways (Supplementary Figure S1C). According to analysis of MF terms, CR related targets are primarily involved in neurotransmitter receptor activity (GO:0030594), protein tyrosine kinase activity (GO:0004713), transmembrane receptor protein tyrosine kinase activity (GO:0004714), G protein-coupled amine receptor activity (GO:0008227), and transmembrane receptor protein kinase activity (GO:0019199) (Figure 2A). These data indicate that CR exerts protective effects on synaptic structure and synaptic function by targeting multiple synapse related targets.

The top 20 most significantly enriched KEGG pathways for the CR targets according to KEGG pathway enrichment analysis are shown in Figure 2B. The main KEGG pathways were the neuroactive ligand-receptor interaction (hsa04080), cAMP signaling pathway (hsa04024), AGE-RAGE signaling pathway in diabetic complications (hsa04933), EGFR tyrosine kinase inhibitor resistance (hsa01521), serotonergic synapse (hsa04726), nitrogen metabolism (hsa00910), endocrine resistance (hsa01522), calcium signaling pathway (hsa04020) and HIF-1 signaling pathway (hsa04066). These results revealed that CR targets are involved in neuronal survival and neuron growth related signals.

### Anti-IS Targets of CR and Their Functional Classifications

**FIGURE 3:**
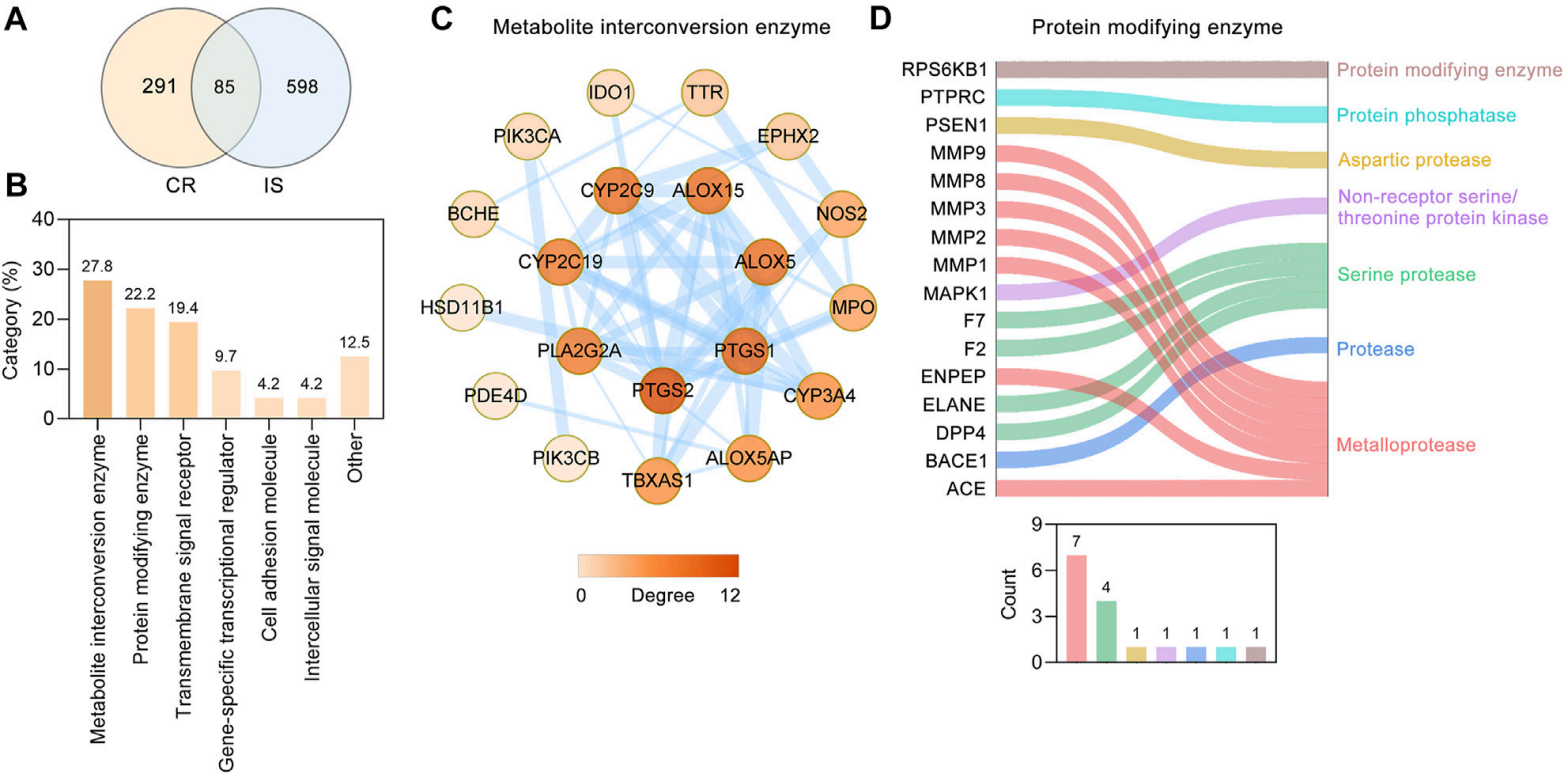
Protein functional classification of targets of CR against IS. **(A)** Venn diagram showing overlapping targets between the CR and IS targets. **(B)** Protein functional classification of 85 common targets. Numbers above bars represent the percentage of the protein in the given functional class. **(C)** PPI network of 20 targets involved in metabolite interconversion enzyme (PC00262). Higher the degree of a node, the darker the color of the node. The edges thickness is proportional to the combined score. **(D)** Sankey diagram of 16 targets involved in protein modifying enzyme (PC00260). The bar chart below the sankey diagram represents the number of targets involved in each classification.

Through retrieving the PALM-IST database (Mandloi and Chakrabarti, 2015) and GeneCards database (Stelzer et al., 2016), altogether, 683 IS-related targets were obtained. To further analyze the underlying mechanism of CR against IS, 85 targets (Table 2) were obtained based on the intersection of CR targets and IS-related targets (Figure 3A). Based on their cellular functions, these 85 targets involved in the anti-IS effect of CR were categorized into seven different classes (Figure 3B). Metabolite interconversion enzyme (PC00262, 27.8%), protein modifying enzyme (PC00260, 22.2%) and transmembrane signal receptor (PC00197, 19.4%) ranked among the top three in terms of the all classes. 20 targets involved in metabolite interconversion enzyme, and these targets formed a complex PPI network (20 nodes and 54 edges) (Figure 3C). Arachidonate 15-lipoxygenase (ALOX15), arachidonate 5-lipoxygenase (ALOX5), CYP2C19, CYP2C9, CYP3A4, IDO1, MPO, PTGS1 and PTGS2 are oxygenases. Among the 16 targets identified as protein modifying enzymes, ACE, ENPEP, MMP1, MMP2, MMP3, MMP8 and MMP9 are metalloproteases, DPP4, ELANE, F2 and F7 are serine proteases (Figure 3D). The above data suggested that CR can stabilize intracellular proteins and the extracellular matrix by regulating various enzymes.

**TABLE 2:** The target information of CR against IS.

Number	Gene ID	Gene symbol	Description
1	5243	ABCB1	ATP binding cassette subfamily B member 1
2	1636	ACE	angiotensin I converting enzyme
3	134	ADORA1	adenosine A1 receptor
4	135	ADORA2A	adenosine A2a receptor
5	153	ADRB1	adrenoceptor beta 1
6	154	ADRB2	adrenoceptor beta 2
7	185	AGTR1	angiotensin II receptor type 1
8	213	ALB	albumin
9	246	ALOX15	arachidonate 15-lipoxygenase
10	240	ALOX5	arachidonate 5-lipoxygenase
11	241	ALOX5AP	arachidonate 5-lipoxygenase activating protein
12	351	APP	amyloid beta precursor protein
13	23,621	BACE1	beta-secretase 1
14	590	BCHE	butyrylcholinesterase
15	6046	BRD2	bromodomain containing 2
16	759	CA1	carbonic anhydrase 1
17	760	CA2	carbonic anhydrase 2
18	1268	CNR1	cannabinoid receptor 1
19	1387	CREBBP	CREB binding protein
20	3576	CXCL8	C-X-C motif chemokine ligand 8
21	1585	CYP11B2	cytochrome P450 family 11 subfamily B member 2
22	1557	CYP2C19	cytochrome P450 family 2 subfamily C member 19
23	1559	CYP2C9	cytochrome P450 family 2 subfamily C member 9
24	1576	CYP3A4	cytochrome P450 family 3 subfamily A member 4
25	1803	DPP4	dipeptidyl peptidase 4
26	1909	EDNRA	endothelin receptor type A
27	1956	EGFR	epidermal growth factor receptor
28	1991	ELANE	elastase, neutrophil expressed
29	2028	ENPEP	glutamyl aminopeptidase
30	2053	EPHX2	epoxide hydrolase 2
31	2099	ESR1	estrogen receptor 1
32	2147	F2	coagulation factor II, thrombin
33	2149	F2R	coagulation factor II thrombin receptor
34	2152	F3	coagulation factor III, tissue factor
35	2155	F7	coagulation factor VII
36	3091	HIF1A	hypoxia inducible factor 1 subunit alpha
37	3290	HSD11B1	hydroxysteroid 11-beta dehydrogenase 1
38	3350	HTR1A	5-hydroxytryptamine receptor 1A
39	3356	HTR2A	5-hydroxytryptamine receptor 2A
40	3383	ICAM1	intercellular adhesion molecule 1
41	3620	IDO1	indoleamine 2,3-dioxygenase 1
42	3553	IL1B	interleukin 1 beta
43	3674	ITGA2B	integrin subunit alpha 2b
44	3683	ITGAL	integrin subunit alpha L
45	3689	ITGB2	integrin subunit beta 2
46	3725	JUN	Jun proto-oncogene, AP-1 transcription factor subunit
47	3791	KDR	kinase insert domain receptor
48	5594	MAPK1	mitogen-activated protein kinase 1
49	4233	MET	MET proto-oncogene, receptor tyrosine kinase
50	4312	MMP1	matrix metallopeptidase 1
51	4313	MMP2	matrix metallopeptidase 2
52	4314	MMP3	matrix metallopeptidase 3
53	4317	MMP8	matrix metallopeptidase 8
54	4318	MMP9	matrix metallopeptidase 9
55	4353	MPO	Myeloperoxidase
56	4780	NFE2L2	nuclear factor, erythroid 2 like 2
57	4843	NOS2	nitric oxide synthase 2
58	4851	NOTCH1	notch receptor 1
59	142	PARP1	poly(ADP-ribose) polymerase 1
60	5144	PDE4D	Phosphodiesterase 4D
61	5290	PIK3CA	phosphatidylinositol-4,5-bisphosphate 3-kinase catalytic subunit alpha
62	5291	PIK3CB	phosphatidylinositol-4,5-bisphosphate 3-kinase catalytic subunit beta
63	5320	PLA2G2A	Phospholipase A2 group IIA
64	5465	PPARA	peroxisome proliferator activated receptor alpha
65	5468	PPARG	peroxisome proliferator activated receptor gamma
66	5663	PSEN1	presenilin 1
67	5742	PTGS1	prostaglandin-endoperoxide synthase 1
68	5743	PTGS2	prostaglandin-endoperoxide synthase 2
69	5788	PTPRC	protein tyrosine phosphatase receptor type C
70	5970	RELA	RELA proto-oncogene, NF-kB subunit
71	5972	REN	renin
72	6198	RPS6KB1	ribosomal protein S6 kinase B1
73	6401	SELE	selectin E
74	5054	SERPINE1	serpin family E member 1
75	6462	SHBG	sex hormone binding globulin
76	6532	SLC6A4	solute carrier family 6 member 4
77	6714	SRC	SRC proto-oncogene, non-receptor tyrosine kinase
78	6774	STAT3	signal transducer and activator of transcription 3
79	6916	TBXAS1	thromboxane A synthase 1
80	7046	TGFBR1	transforming growth factor beta receptor 1
81	7099	TLR4	toll like receptor 4
82	706	TSPO	translocator protein
83	7276	TTR	transthyretin
84	7412	VCAM1	vascular cell adhesion molecule 1
85	7422	VEGFA	vascular endothelial growth factor A

**FIGURE 4:**
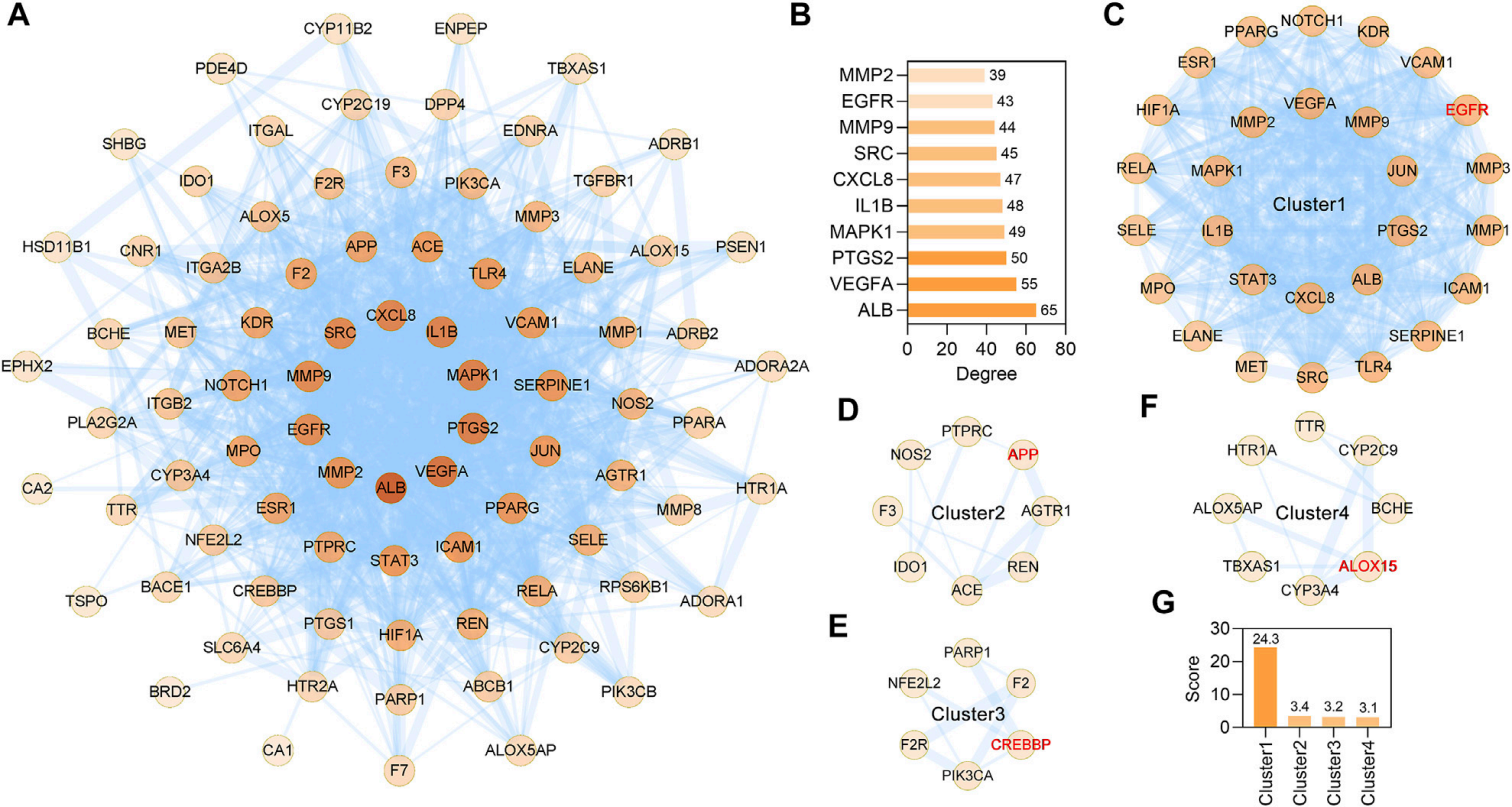
MCODE network clustering analysis of common targets between the CR and IS. **(A)** The PPI network was constructed for the 85 potential targets of CR against IS. The darker the node, the greater the degree. The edges thickness is proportional to the combined score. **(B)** The top 10 core targets were extracted from **(A)**. **(C–F)** The four tightly connected network clusters were identified by MCODE algorithm. The seed node of each cluster is indicated by red font. **(G)** Comparison of MCODE scores for different clusters.

STRING database (version 11.5) based PPI analysis (Szklarczyk et al., 2019) was performed to investigate the relationship between the 85 anti-IS targets. The PPI network contained a total of 85 nodes and 920 edges, and the average node degree was 21.6 (Figure 4A). When the targets were sorted by degree, ALB, VEGFA, PTGS2, MAPK1, IL1B, CXCL8, SRC, MMP9, EGFR and MMP2 were identified as the core targets (Figure 4B). Furthermore, through MCODE analysis (Bader and Hogue, 2003), four functional clusters that may be the most relevant to the anti-IS mechanism of CR were identified from the 85 anti-IS targets (Figures 4C–F). Cluster one contained 28 nodes and 328 edges with a score of 24.3 (Figures 4C,G). The seed node of this cluster was EGFR (Figure 4C), which plays a major role in cell growth, proliferation, survival and differentiation. Cluster one involved targets that are mainly involved in the inflammatory response (GO:0006954), and MMP9, PTGS2, CXCL8, TLR4, SERPINE1, IL1B may be potential therapeutic targets in the effect of CR on the inflammatory response. CXCL8, IL1B, MMP9 and PTGS2 are also core targets, which indicates the importance of the inflammatory response in the therapeutic effect of CR on IS. Lowest energy docking model based molecular docking analysis was applied to validate the binding of the core targets (CXCL8, IL1B, MMP9, PTGS2) and CR phytochemicals. The results showed that all the core targets were well combined with the corresponding CR phytochemicals (Table 3). Among these targets, senkyunolide I showed the highest binding energy with PTGS2, with score values of −5.53 kcal/ mol; rulic acid showed the highest binding energy with PTGS2 and MMP9, with score values of −5.11 and −7.04 kcal/ mol, respectively; coniferyl ferulate showed the highest binding energy with MMP9, with score values of −7.91 kcal/ mol; nocnidilide and lvistilide A showed the highest binding energy with IL1B and CXCL8, respectively.

**TABLE 3:** Molecular docking of the core targets with its corresponding CR phytochemicals.

Ligands	Targets	PDB ID	Score (kcal/mol)
Senkyunolide I	PTGS2	5F19	−5.53
Ferulic acid	PTGS2	5F19	−5.11
Z-ligustilide	PTGS2	5F19	−4.54
Butylidenephthalide	PTGS2	5F19	−4.54
Neocnidilide	PTGS2	5F19	−4.26
Neocnidilide	IL1B	5R85	−6.43
Senkyunolide A	IL1B	5R85	−6.32
Levistilide A	CXCL8	1ILQ	−6.59
Coniferyl ferulate	MMP9	2OW0	−7.91
Ferulic acid	MMP9	2OW0	−7.04
L-Tryptophan	MMP9	2OW0	−6.38
Vanillic acid	MMP9	2OW0	−6.35
Caffeic acid	MMP9	2OW0	−6.15

Cluster two contained eight nodes and 12 edges with a score of 3.4 (Figures 4D,G) and was primarily involved in blood circulation (GO:0008015). The seed node of this cluster was APP, which encodes a cell surface receptor and transmembrane precursor protein that is cleaved by secretases to form various peptides (Figure 4D). Cluster 3 contained 6 nodes and 8 edges with a score of 3.2 (Figures 4E,G). The seed node of this cluster was CREB binding protein (CREBBP) (Figure 4E). CREBBP (Gouveia et al., 2017), NFE2L2 (Kidani et al., 2020) and PARP1 (Liu et al., 2021) are known for their roles in the pathogenesis of stroke and post-stroke neurovascular remodeling and functional recovery. Cluster 4 contained 8 nodes and 11 edges with a score of 3.1 (Figures 4F,G) and ALOX5AP, ALOX15, CYP2C9, CYP3A4, TBXAS1, HTR1A, TTR and BCHE are oxidative stress related targets. ALOX15 is the seed node of this cluster (Figure 4F). Of note, ALOX15 is up-regulated following IS and contributes to neuronal cell death and hemorrhagic transformation (Gaberel et al., 2019). These data suggest that CR has anti-inflammation, anti-oxidative stress, anti-cell death activities and improves blood circulation to treat IS.

### Key Phytochemicals of CR in Treating IS

**FIGURE 5:**
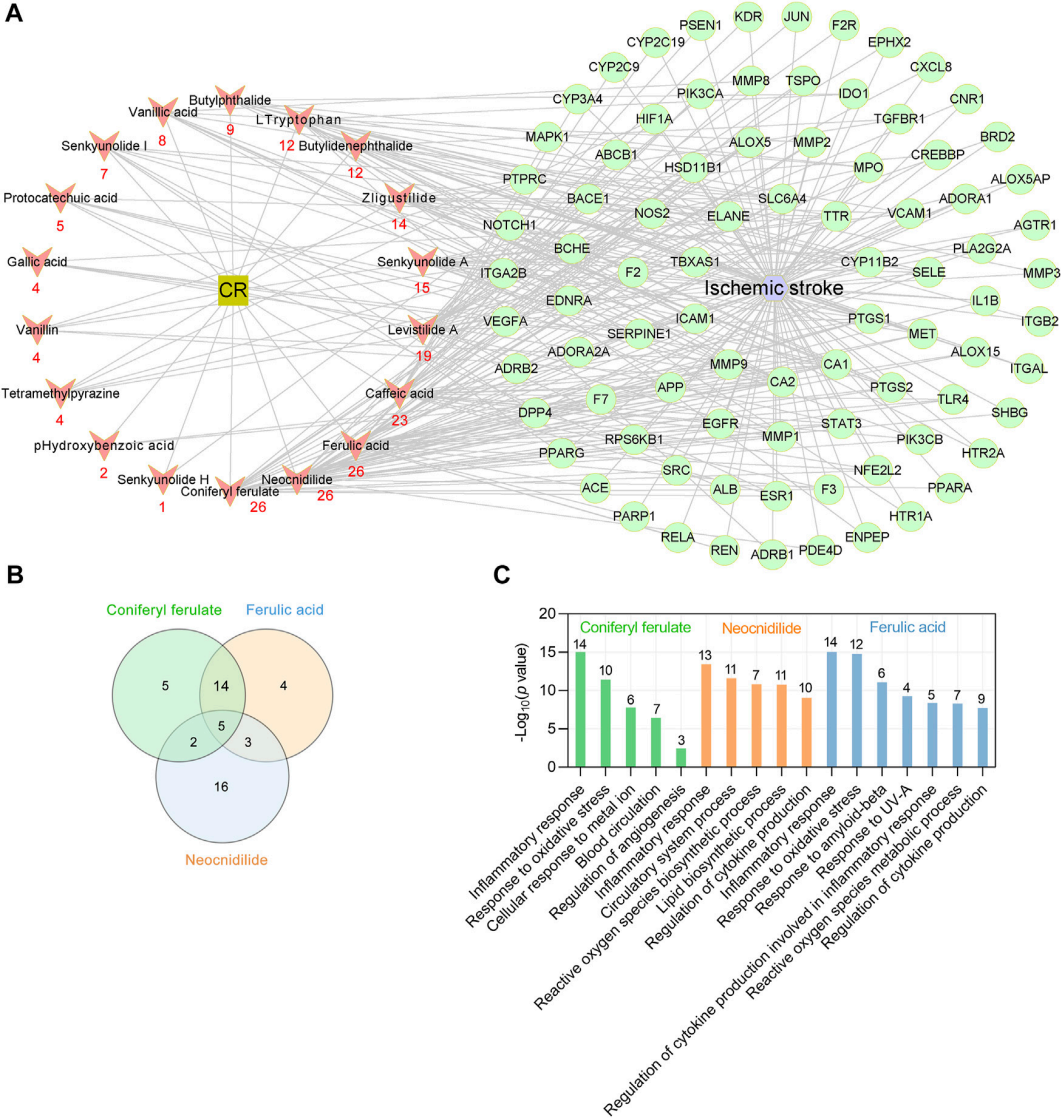
Key phytochemicals of CR in treating IS. **(A)** CR main phytochemicals target-IS target network. Red nodes represent the main phytochemicals in CR, and red numbers below these nodes represent the number of CR targets against IS. Green circular nodes represent 85 common targets between the CR and IS. **(B)** Venn plot showing the number of overlapping potential targets among the three key ingredients. **(C)** Enriched GO biologic process for top three key phytochemicals -related targets. Numbers above bars are the number of targets enriched in terms.

To identify the key phytochemicals of CR in treating IS, a CR main component target-IS target network was constructed using Cytoscape software (version 3.7.1) (Shannon et al., 2003). The interaction network comprised 18 active phytochemicals of CR and 85 corresponding targets (shown as gene symbols) (Figure 5A). The average number of targets per CR component was 12.1, and the mean of degree of phytochemicals per target was 2.6. These results fully reflect the multicomponent and multitarget characteristics of CR in the treatment of IS. The results showed that coniferyl ferulate, neocnidilide and ferulic acid have the most targets (degree = 26), followed by caffeic acid (degree = 23), levistilide A (degree = 19), senkyunolide A (degree = 15) and z-ligustilide (degree = 14) (Figure 5A). The chemical structures of CR phytochemicals with top 10 anti-IS targets are presented in Supplementary Figure S2. Coniferyl ferulate acts on four core targets, including EGFR, MMP2, MMP9 and SRC. Neocnidilide acts on two core targets (IL1B and PTGS2) and ferulic acid acts on four core targets (EGFR, MMP2, MMP9 and PTGS2). Interestingly, coniferyl ferulate, neocnidilide and ferulic acid have many common targets (Figure 5B). Furthermore, they all act on the inflammatory response (GO:0006954) and oxidative stress (GO:0006979). Coniferyl ferulate and ferulic acid both regulate cytokine production (Figure 5C). Thus, coniferyl ferulate, neocnidilide and ferulic acid are the key phytochemicals of CR in treating IS.

### Potential Synergistic Effects of CR Phytochemicals in Treating IS

As shown in Figure 5, the phytochemicals of CR have the same targets the different targets, suggesting that CR may act on different pathological processes of IS. Therefore, we used the clusterProfiler R package to determine the potential synergistic effects of CR phytochemicals in treating IS according to the 85 IS associated targets of CR by analyzing their enrichment for GO BP terms and KEGG pathways.

**FIGURE 6:**
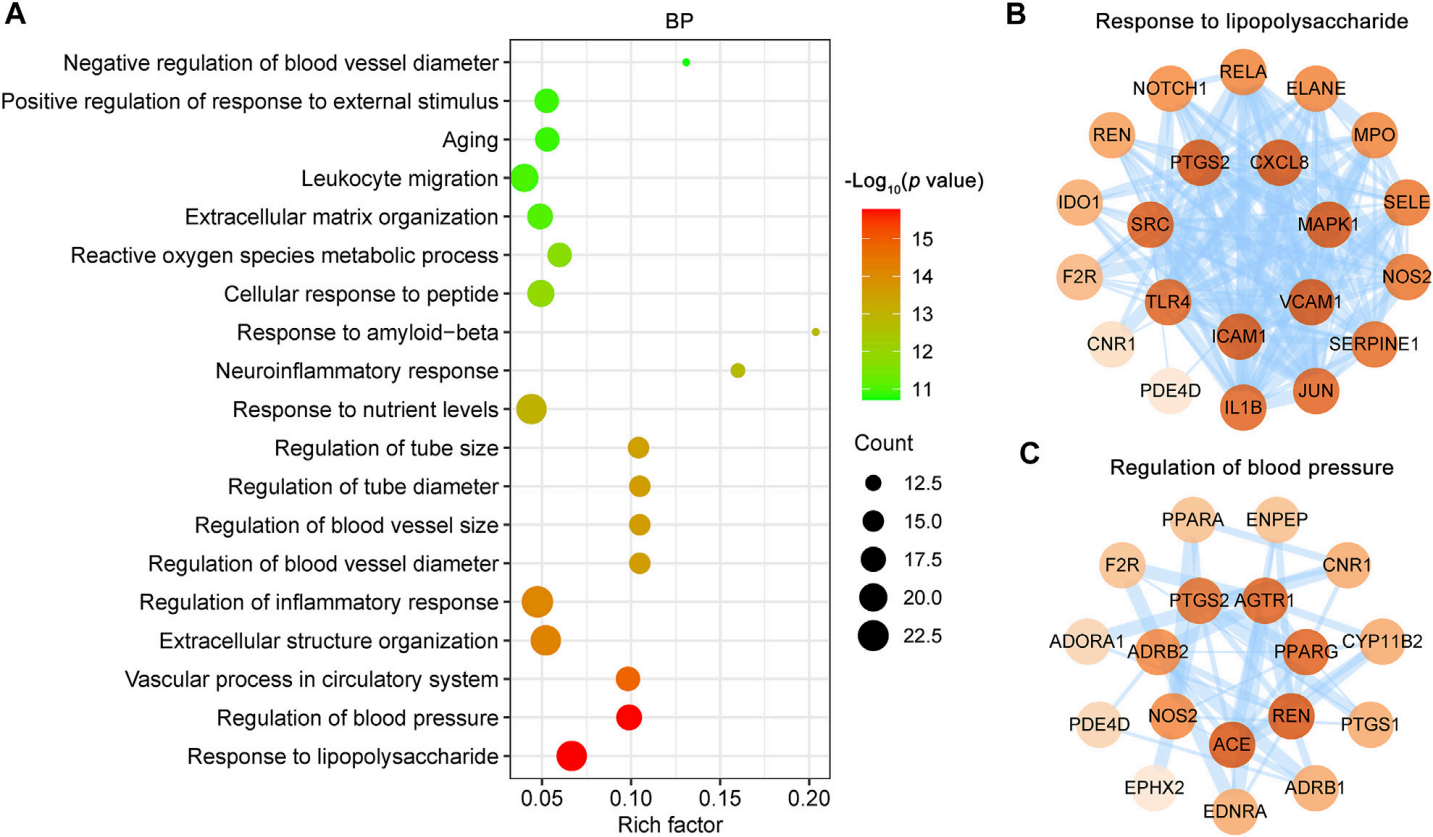
GO biological process (BP) enrichment analysis of targets of CR against IS. **(A)** Bubble plot of representative GO enrichment terms of common targets between the CR and IS. Only functional BP terms passing adjust p value threshold <0.05 were considered. X-axis, rich factor; bubble size, the number of genes enriched; bubble color, *p* value. **(B, C)** PPI network of targets involved in response to lipopolysaccharide (GO:0032496) and regulation of blood pressure (GO:0008217). Higher the degree of a node, the darker the color of the node. The edges thickness is proportional to the combined score.

The 85 shared targets of CR and IS are significantly involved in response to LPS (GO:0032496), regulation of blood pressure (GO:0008217), vascular process in circulatory system (GO:0003018), extracellular structure organization (GO:0043062), regulation of inflammatory response (GO:0050727) and so on (Figure 6A). The endotoxin, LPS, triggers inflammatory cascade, complement activation, prooxidative stress, and tissue destruction. 22 IS associated CR targets are involved in the response to LPS (GO:0032496) and form a PPI network that includes 21 nodes and 137 edges (Figure 6B). Notably, ICAM1, VCAM1, MAPK1, CXCL8 and PTGS2 play a key role in this PPI network (Figure 6B). Infection is the leading cause of death in patients following stroke. Acute IS is followed by profound immunoreactions, including an inflammatory response and subsequent stroke-induced immunodepression syndrome (SIDS). SIDS further promotes infection complications, such as the most common pneumonia, and brain damage. The regulation of inflammatory response (GO:0050727), neuroinflammation response (GO:0150076), reactive oxygen species metabolic process (GO:0072593) and leukocyte migration (GO:0050900) are all important for the treatment of SIDS.

An abrupt increase in blood pressure is the most common clinical symptom of acute IS. As blood pressure changes during acute IS vary with the time of onset and between patients, the optimal blood pressure management after acute IS remains a challenge. As shown (Figure 6A), regulation of blood pressure (GO:0008217) was the second most enriched term, with the PPI network including 18 nodes and 46 edges (Figure 6C). Additionally, the vascular process in circulatory system (GO:0003018), regulation of blood vessel diameter (GO:0097746) and regulation of blood vessel size (GO:0050880) (Figure 6A) all contribute to blood pressure management. According to these results, infection prevention and blood pressure regulation are the two important synergistic effects of CR phytochemicals in treating IS.

### CR Improves Cerebral Ischemic Injury and Its Phytochemicals Entering the Brain

**FIGURE 7:**
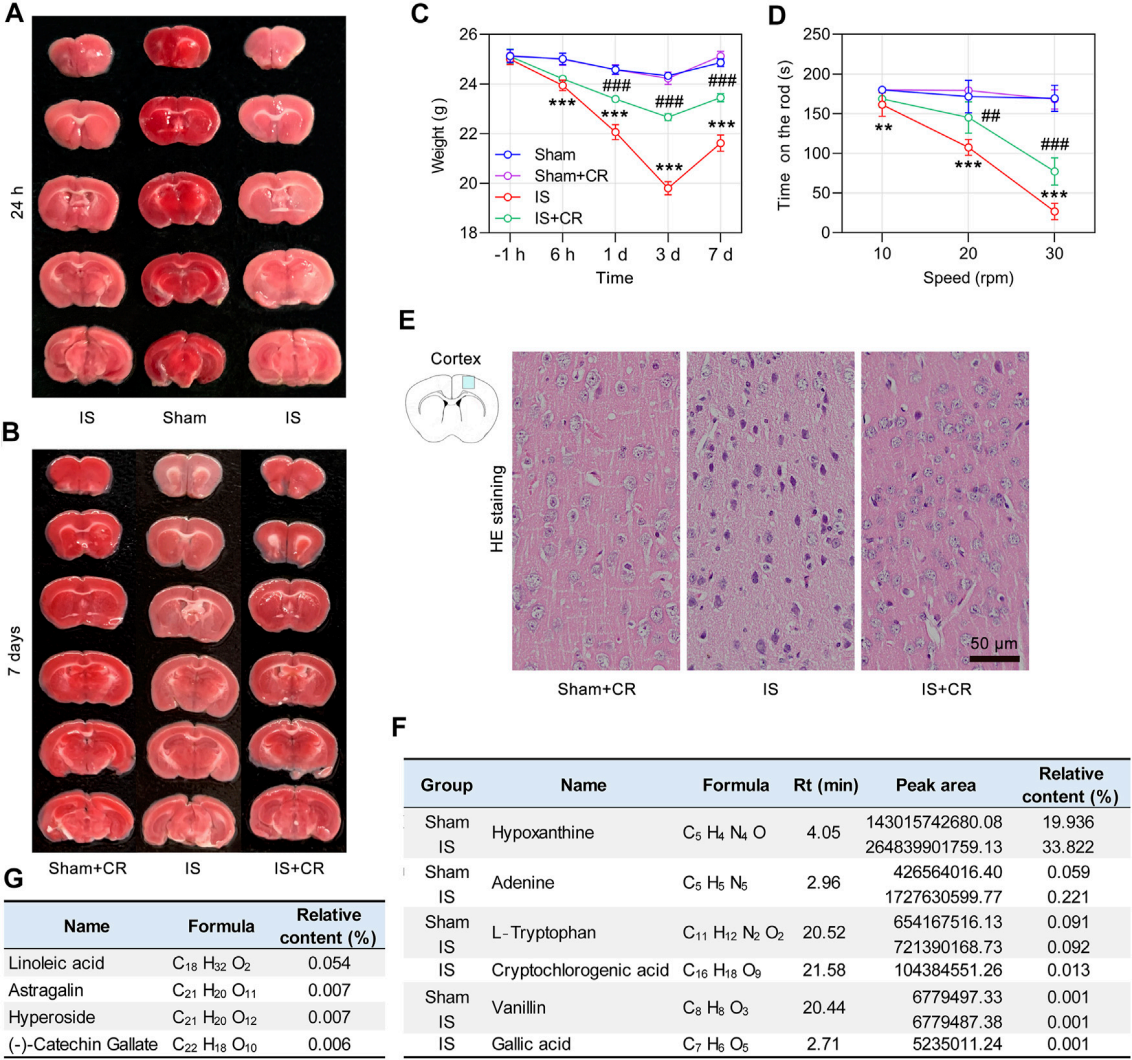
CR treatment significantly ameliorates cerebral ischemic injury. Representative images of TTC-stained brain slices obtained 24 h **(A)**, or 7 days **(B)** after global cerebral ischemia surgery. **(C)** Body weight changes were monitored over a 7-day period. **(D)** The time on the rod in the rotarod test on day 3 (*n* = 6/group). ** *p* < 0.01, *** *p* < 0.001 *vs*. Sham; ## *p* < 0.01, ### *p* < 0.001 IS + CR *vs*. IS. **(E)** Representative images of HE staining of the cortex after 7 days of CR treatment. Normal cortical neurons revealed normal cells with rich cytoplasm and round and slightly stained nucleus with relatively large and clear nucleolus formation. Scale bar = 50 µm. **(F)** The phytochemicals identified by UHPLC/MS/MS in the brains of Sham and IS mice that were orally administered CR. **(G)** Four components detected only in the brains of CR treated IS mice.

Twenty-four hours after establishing the global cerebral ischemia model, cerebral infarction was observed in IS mice. The infarcted brain tissue was pale, and normal brain tissue was red (Figure 7A). The cerebral ischemia injury was improved after 7 days of gavage treatment of CR (1,300 mg/ kg/ day) (Figure 7B). Intragastric treatment with CR can improve weight loss in IS mice (Figure 7C). To assess cerebral ischemia-induced impairment of motor coordination, the rotarod test was performed. Compared with Sham and IS + CR mice on day 3, the IS mice showed a significant reduction in retention time (Figure 7D). Neuronal morphology of the mice cerebral cortex was observed 24 h after cerebral ischemia surgery by HE staining (Figure 7E). Cortical neurons in Sham mice after CR treatment were arranged regularly, and the structures of neurons were clearly with round, large and regular nuclei. Most of the neurons of IS mice were arranged disorderly, and the nucleus was pyknotic or severely shrunken. These results indicated that treatment with CR may improve brain injuries caused by cerebral ischemia.

After 7 days of CR treatment, less cellular damage was observed. Studying components of CR in the brain may help to understand its cerebral protections. Therefore, an UHPLC/MS/MS method was employed to detect the relative contents of CR components in the brain samples from CR treated Sham and IS mice as shown in Supplementary Figures S3A,B. A total of 148 chemical constituents were screened out. In brain samples of CR treated Sham mice, L-tryptophan (0.091%) and vanillin (0.001%) were detected, suggesting they enter the brain tissue under physiological condition. In CR treated IS mice L-tryptophan (0.092%), GA (0.001%), vanillin (0.001%) and CA (0.013%) were detected (Figure 7F). In the UHPLC/MS/MS data, we also found the increased contents of hypoxanthine (33.82%) and adenine (0.221%) in brains of IS mice (Figure 7F). Hypoxanthine acts an endogenous monoamine oxidase (MAO) inhibitor. The serum level of hypoxanthine in the patients with IS was significantly decreased (Wang D. et al., 2017). Adenosine is a key nucleoside and regulates energy homeostasis to affect the function of the brain. Under IS condition, GA and CA entered the brain (Figure 7F). GA, a well-known antioxidant compounds, has neuroprotective actions in different models of neurodegeneration, neurotoxicity, and oxidative stress (Daglia et al., 2014). CA could attenuate LPS-induced inflammatory symptoms and oxidative stress (Zhao et al., 2020). Additionally, it was found that linoleic acid, astragalin, hyperoside and (-)-Catechin Gallate (CG) detected only in the brains of IS mice (Figure 7G). Linoleic acid, an essential polyunsaturated fatty acid, is required for normal growth and development (Taha, 2020). Astragalin (Riaz et al., 2018), hyperoside (Chen et al., 2021) and CG (Lee et al., 2020) are well known for their activities such as anti-inflammation, anti-oxidant and neuroprotection. From above data, anti-oxidant and anti-inflammation may be two important aspects of CR’s cerebral protections in IS, which needs to be further verified.

## Discussion

In this study we used a data processing method that combines network pharmacology analysis with the important pathophysiological processes of IS, and uncovered the key phytochemicals and biological functions of CR and its underlying mechanisms in the treatment of IS. A total of 18 bioactive phytochemicals of CR corresponding to 85 anti-IS targets were obtained, and coniferyl ferulate, neocnidilide and ferulic acid were identified as the key phytochemicals of CR against IS. CR exerts protective effects on neurons mainly by acting on targets related to synaptic structure, synaptic function, neuronal survival and neuronal growth. CR also has the activities of anti-inflammation, anti-oxidative stress, anti-cell death and improves blood circulation. Infection and an abrupt increase in blood pressure are common complications of IS. In this research, we found that infection prevention and blood pressure regulation are the two most important synergistic effects of CR phytochemicals in treating IS.

Although network pharmacology approach contributes to collect active phytochemicals and pharmacological actions of herbs or Chinese medicine prescriptions, this approach also has many limitations. One important issue is the reliability of the data source. Normally, vast majority of network pharmacology studies used public databases to screen the active phytochemicals in traditional Chinese medicine. This would result in a large number of non-specific phytochemicals to be screened out. For example, seven active phytochemicals in CR have an oral bioavailability (OB) ≥ 30% and drug likeness (DL) ≥ 0.18 according to the Traditional Chinese Medicine Systems Pharmacology Database and Analysis Platform (TCMSP) (Ru et al., 2014). However, this screening method does not consider the content of each component. Among these phytochemicals, sitosterol exists widely in 179 kinds of herbs and mandenol exists in 35 kinds of herbs (data from HERB, http://herb.ac.cn/, date of search: October 20, 2021) (Fang et al., 2021). Obviously, compounds that are widely distributed in a variety of herbs and have good pharmacological properties are more likely to be identified as active phytochemicals. Unlike the conventional approach, the present study obtained data on active CR component, including aromatic acids, phthalides and tetramethylpyrazine from recent UHPLC-MS/MS-based studies (Yin et al., 2019; Wang et al., 2020). In stark contrast, butylidenephthalide, neocnidilide, senkyunolide A and z-ligustilide are only present in 3, 6, 10, and 13 herbs, respectively, including CR (data from HERB, date of search: October 20, 2021). There are differences in the phytochemicals of the same Chinese medicine with different harvest time and producing area. The contents of CR phytochemicals were analyzed using 36 CR samples from six production origins. It is necessary to design an algorithm to quantitatively evaluate the impact of component content on drug efficacy in a future study. Thus, the reliability of the source of CR component data and the follow-up analysis are significantly increased in this study. However, identification of key phytochemicals only through network pharmacological analysis has certain limitations, such as the complex dose-effect relationship and toxicological evaluation was not considered. Notably, most research based on network pharmacology is still static network analysis, which contradicts the dynamic changes of disease occurrence and development. Moreover, chemical changes that occur during various drug manufacturing processes are not considered in this study.

Among the phytochemicals of CR, 18 active phytochemicals meet with RO5 (Lipinski, 2004) and correspond to 376 targets. The intersection of drugs and disease targets can lead to inflated pathway enrichment analysis. Therefore, before analyzing the anti-IS effects of CR, it was necessary to obtain a more comprehensive understanding of the biological functions of CR-related targets. Unexpectedly, most CR targets are synaptic membrane proteins and involve multiple synapses. Synapses, as an essential unit for neuronal communication in the brain, and synaptic plasticity can accelerate functional recovery after IS (Clarkson et al., 2010; Clarkson et al., 2011). Neurons in the center of the ischemic region undergo liquefactive necrosis, during which the body and axons of neurons disappear (Park et al., 2013; Nguyen et al., 2016). IS can lead to neurological deficits (Kuklina et al., 2012; Esenwa and Gutierrez, 2015), and significantly enriched neuroactive ligand–receptor interaction signaling pathway (hsa04080) is directly related to neurological function. Mounting evidence suggests that the cAMP signaling pathway plays important biological roles in IS (Xin et al., 2020). Astaxanthin activates the cAMP/PKA signaling pathway to promote axonal regeneration around the infarction and improve motor function (Wang et al., 2019). Apoptosis is an important cause of neuronal death after IS, and hypoxia and calcium overload can induce cell apoptosis (Radak et al., 2017). KEGG pathway enrichment analysis revealed that CR can affect the apoptosis pathway by acting on the calcium signaling pathway and HIF-1 signaling pathway. In addition to exerting neuroprotective effects, CR affects multiple signaling pathways and structures associated with cerebral circulation, such as the HIF-1 signaling pathway, the VEGF signaling pathway and gap junctions.

The CR main component target-IS target network indicated that coniferyl ferulate, neocnidilide, ferulic acid, caffeic acid, levistilide A, senkyunolide A and z-ligustilide are likely to become key phytochemicals for CR against IS. Coniferyl ferulate possesses multiple pharmacological activities such as antibacterial (Chou et al., 2006), antioxidant (Li et al., 2007) and vasodilating effects (Dai et al., 2017). Treatment with coniferyl ferulate (50 or 100 mg/ kg for 14 days) exerts antidepressant effect by acting on NMDAR-CaMKII-MAPKs signaling pathway, oxidative stress, and mitochondrial apoptotic pathways (Gong et al., 2020). The content of z-ligustilide in CR was the highest and has vasodilatation (Cao et al., 2006). Neocindilide exhibits anti-inflammatory and antioxidant activities. The role of ferulic acid included anti-thrombosis (Hsieh et al., 2002). 5-lipoxygenase (ALOX5) is a key enzyme metabolizing arachidonic acid to produce leukotrienes. Caffeic acid is not only a specific inhibitor of ALOX5 but also possesses antioxidant and anti-inflammatory properties (Habtemariam, 2017). An animal study demonstrated that caffeic acid (30 or 50 mg/ kg) ameliorates global cerebral ischemia-reperfusion injury in rats by inhibiting ALOX5 (Liang et al., 2015). In addition, caffeic acid can inhibit platelet aggregation induced by collagen (Hsiao et al., 2007; Park, 2009). Currently, levistilide A is a potential P-gp modulator and used for treating cancer. Senkyunolide A and z-ligustilide are used in treating inflammation associated with cerebrovascular diseases (Or et al., 2011). In this study L-tryptophan and vanillin were detected in brains of Sham and IS mice, while linoleic acid, astragalin, hyperoside and CG detected only in the brains of IS mice. L-tryptophan, a precursor of serotonin, is used to improve the mood of healthy individuals (Kikuchi et al., 2021) and depressed individuals (Alvarez-Mon et al., 2021). The significantly decreased L-tryptophan in the brain was reported in acute ischemic stroke (Ormstad et al., 2013). Vanillin is reported as an anti-oxidative, anti-apoptotic, anti-inflammatory, and neuroprotective compound (Bezerra-Filho et al., 2019). Furthermore, vanillin has the ability of maintaining the integrity of the BBB (Lan et al., 2019). Linoleic acid was reported significantly increase the axonal outgrowth (Hennebelle et al., 2020). An inverse association between linoleic acid levels in circulation (Ye et al., 2020) and adipose tissue (Veno et al., 2018) with the risk of IS was reported. Astragalin (Riaz et al., 2018), hyperoside (Chen et al., 2021) and CG (Lee et al., 2020) are well known for their activities such as anti-inflammation, anti-oxidant and neuroprotection. Although these compounds have been detected in the mouse brain, we do not know how these components were produced from CR in the mouse body.

Inflammatory response is a key component in the pathophysiology of IS (Chen et al., 2017). Cluster one was identified to be involved in the inflammatory response, and its targets CXCL8, IL1B, MMP9 and PTGS2 are the core targets of CR in the treatment of IS. These core targets are involved in the NF-κB signaling pathway and interleukin-17 (IL-17) signaling pathway. The proinflammatory chemokine CXCL8, also known as interleukin-8 (IL-8), is involved in acute inflammation. Animal experiments have demonstrated that functional suppression of CXCL8 promotes neuroglial activation and inhibits neuroinflammation by regulating the PI3K/Akt/NF-κB signaling pathway (Lv et al., 2019). Functional analysis with DNA microarray also revealed that CXCL8 and TNF are the feature genes of IS and may be valid targets for IS treatment (Zhang et al., 2014). Moreover, the serum CXCL8 level may be a prognostic indicator for IS (He et al., 2018). MMP9 and MMP2 (belong to gelatinase) are activated during IS and can activate numerous proinflammatory agents as chemokine CXCL8, IL1B or TNFα (Kurzepa et al., 2014). As an important prognostic factor for IS, elevated levels of serum MMP9 in the acute phase of IS are associated with increased risk of mortality and major disability (Zhong et al., 2017). Many studies have shown that the level of matrix metalloproteinase (especially MMP9) increases after stroke, and is associated with BBB disruption (Turner and Sharp, 2016). A clinical study showed that the increased serum MMP9 level in acute phase of IS was associated with 3-months cognitive impairment (Zhong et al., 2018). Selective inhibition of gelatinase can be used to treat IS. Administration of MMPs inhibitors (BB-94) before rtPA treatment reduced mortality, suggesting that blocking MMPs activity reduces the risk associated with thrombolysis (Pfefferkorn and Rosenberg, 2003). In the acute phase of IS, IL1B and TNFα promote inflammatory injury and induce cell necrosis or apoptosis (Bi et al., 2015). Reduction of serum IL1B ultimately improve the clinical outcome of patients with IS (Kadri et al., 2020). A previous study showed that PTGS2 (also known as cyclooxygenase-2) produced prostaglandins and thromboxanes, which are important mediators of IS-induced inflammatory cascade (Dong et al., 2019). Protein expression of PTGS2 increased significantly after IS. The specific knockdown of PTGS2 can inhibit NF-κB signaling pathway, thus inhibit apoptosis, promote the proliferation, migration and angiogenesis of endothelial progenitor cells, and protect ischemic stroke mice (Zhou et al., 2019). Furthermore, the inflammatory response after IS can also lead to neuron apoptosis, forming a vicious circle, which in turn aggravates the inflammatory response (Mergenthaler et al., 2004). Overall, our findings indicate that CR can act on the above inflammatory response related core targets in the treatment of IS.

## Conclusions

This study provides a preliminary exploration of the therapeutic effect of CR on ischemic brain injuries and its possible phytochemicals, and coniferyl ferulate, neocnidilide and ferulic acid are identified the key phytochemicals of CR against IS. Its brain protective effects involve anti-inflammation, anti-oxidant, anti-cell death and improving blood circulation. Simultaneously, preventing infection and regulating blood pressure are the two important manifestations of the synergistic effects of CR in treating IS. However, subsequent *in vivo* (oxygen-glucose deprivation model) and *in vitro* experiments need to be further proved. In addition, toxicity and safety studies should be performed before clinical translation. In summary, this study provides a pathophysiologically relevant pharmacological basis for further research on IS.

## Data Availability

The raw data supporting the conclusions of this article will be made available by the authors, without undue reservation.

## Glossary

ALOX15: arachidonate 15-lipoxygenase
APP: amyloid beta precursor protein
BBB: blood-brain barrier
BP: biological process
CC: cellular component
CR: Chuanxiong Rhizome
CREBBP: CREB binding protein
DL: drug likeness
EGFR: epidermal growth factor receptor
GO: Gene ontology
Hacc: hydrogen bond acceptors
Hdon: hydrogen bond donors
ICAs: internal carotid arteries
IL-8: interleukin-8
IS: Ischemic stroke
KEGG: Kyoto Encyclopedia of Genes and Genomes
LogP: lipid-water partition coefficient
MAO: monoamine oxidase
MCAO: middle cerebral artery occlusion
MCODE: Molecular Complex Detection
MF: molecular function
MW: molecule weight
OB: oral bioavailability
PALM-IST: Pathway Assembly from Literature Mining-an Information Search Tool
PPI: protein-protein interaction
Rbon: rotatable bonds
RO5: Lipinski’s rule of five
ROS: reactive oxygen species
TCMSP: Traditional Chinese Medicine Systems Pharmacology Database and Analysis Platform
TTC: Triphenyltetrazolium chloride
tPA: tissue plasminogen activator
UHPLC-MS/MS: ultra-HPLC with triple quadrupole MS
