# Supplementary figures and images for: Key Phytochemicals and Biological Functions of Chuanxiong Rhizoma Against Ischemic Stroke: A Network Pharmacology and Experimental Assessment
Source: Front Pharmacol. 2021 Dec 21;12:758049. doi: 10.3389/fphar.2021.758049 (PMC8724589; doi:10.3389/fphar.2021.758049)

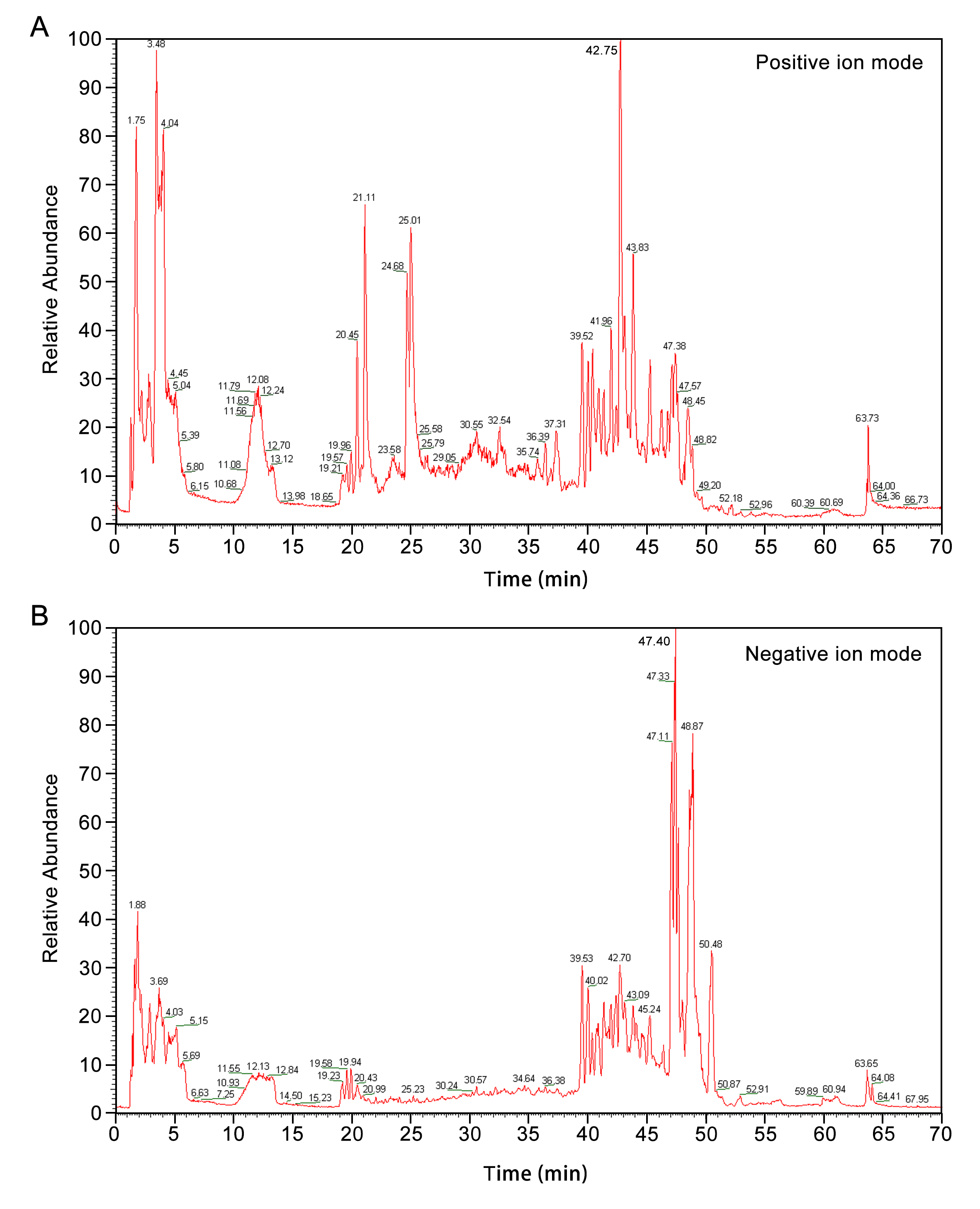

Supplement: Supplementary file 1 [file Image3.TIF]

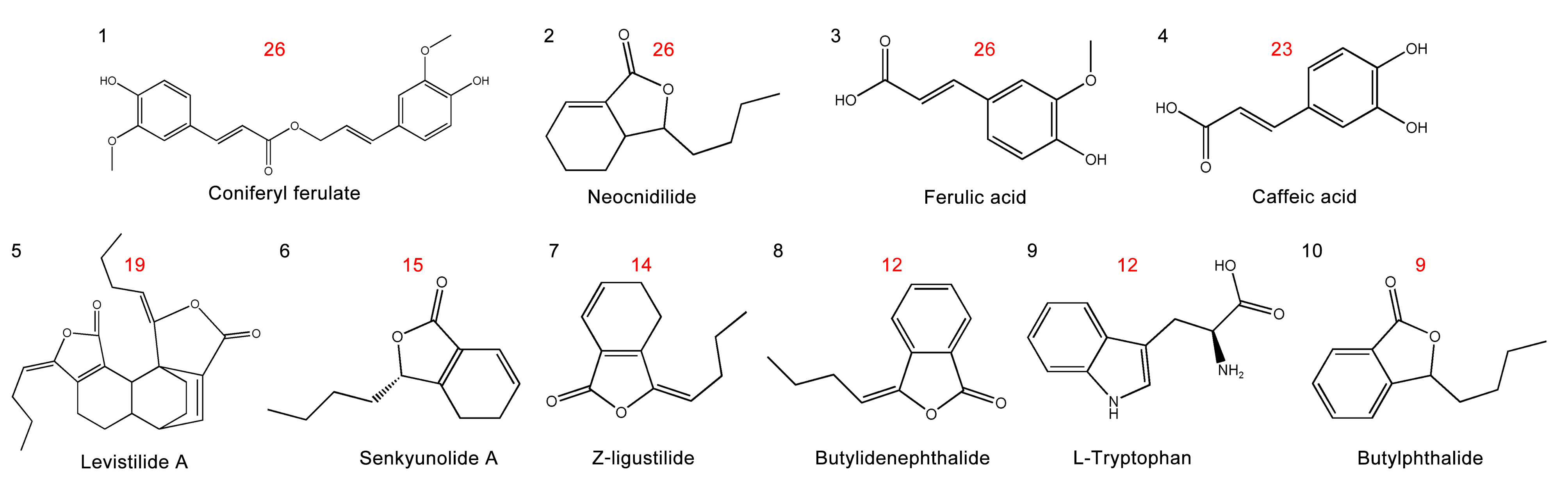

Supplement: Supplementary file 2 [file Image2.TIF]

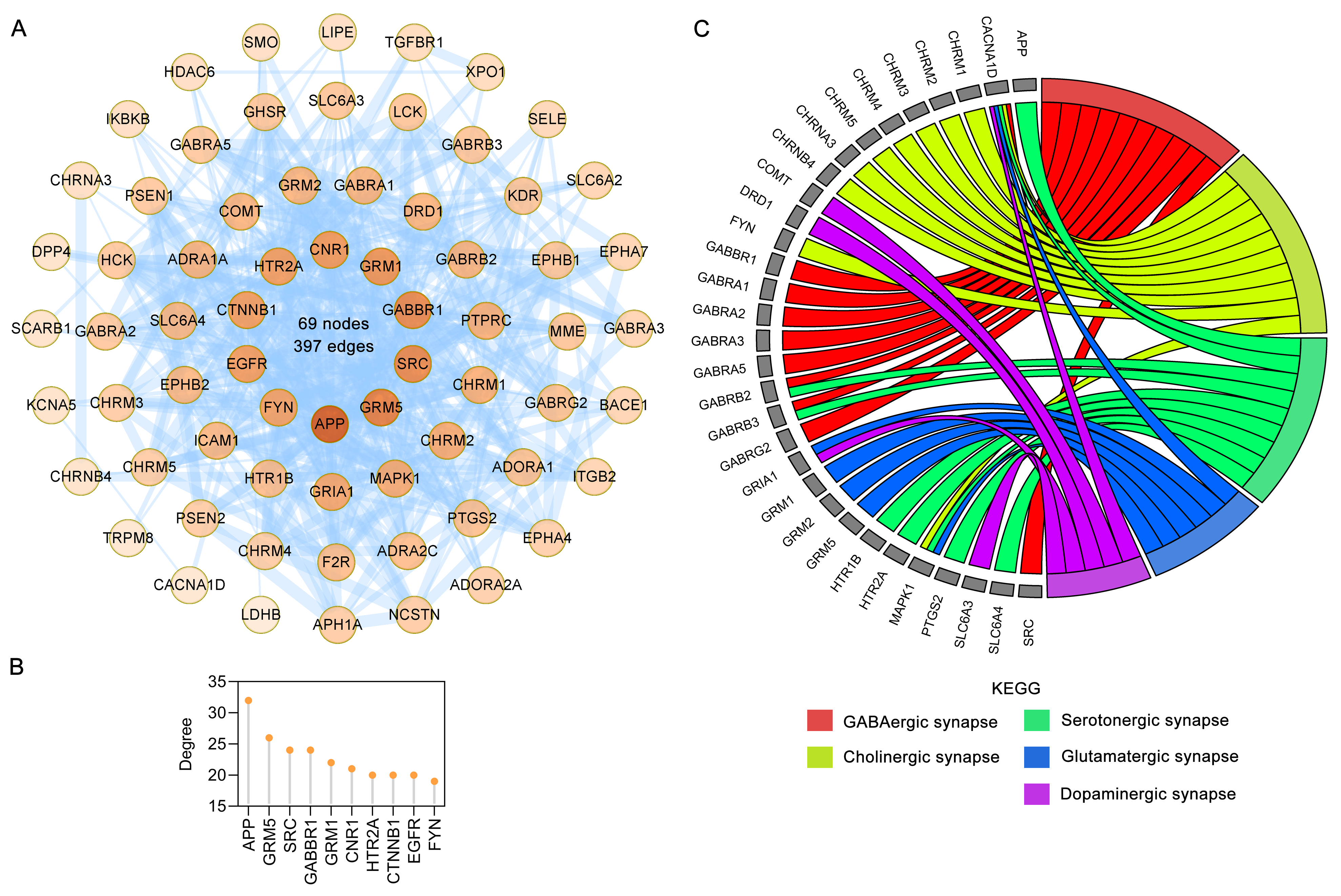

Supplement: Supplementary file 3 [file Image1.TIF]
